# Determinants of Poor Treatment Adherence among Patients with Type 2 Diabetes and Limited Health Literacy: A Scoping Review

**DOI:** 10.1155/2022/2980250

**Published:** 2022-07-04

**Authors:** Nasrin Pourhabibi, Bahram Mohebbi, Roya Sadeghi, Elham Shakibazadeh, Mojgan Sanjari, Azar Tol, Mehdi Yaseri

**Affiliations:** ^1^School of Public Health Tehran University of Medical Sciences, Tehran, Iran; ^2^Cardiovascular Intervention Research Center, Cardio-Oncology Research Center, Rajaie Cardiovascular Medical and Research Center, Iran University of Medical Sciences, Tehran, Iran; ^3^Department of Health Promotion and Education, School of Public Health Tehran University of Medical Sciences, Tehran, Iran; ^4^Department of Internal Medicine, School of Medicine Endocrinology and Metabolism Research Center Afzalipour Hospital, Kerman University of Medical Sciences, Kerman, Iran; ^5^Department of Health Education and Health Promotion, School of Public Health, Tehran University of Medical Sciences, Tehran, Iran; ^6^Department of Epidemiology and Biostatistics, School of Public Health Tehran University of Medical Sciences, Tehran, Iran

## Abstract

Prevention of complications and successful control of diabetes require preventive and therapeutic measures. Patients' nonadherence to medication and diet regimens and healthcare protocols is associated with significant therapeutic and economic consequences. The present scoping review aims to identify determinants of poor treatment adherence among patients with type 2 diabetes and limited health literacy in 2021. This scoping review was conducted in five stages: designing a research question, searching and extracting related studies, selecting related studies, tabulating information, and reporting results. Data were collected from six foreign electronic databases (Embase, Science Direct, PubMed, Google Scholar, Scopus, and Web of Science) and four Iranian electronic databases (MagIran, SID, IranDoc, and IranMedex) using keywords “Type 2 diabetes”, “barriers”, “treatment”, “medication”, “adherence”, “non-adherence”, “limited adherence”, and “limited health literacy” from January 2010 to November 2021. From an initial 146 articles, 18 articles were eligible for review. Eighteen studies involving 3925 patients with T2DM from eight countries were included. The prevalence of nonadherence ranged from 42% to 74.3%. Barriers to treatment adherence, which were common among the articles, included economic problems, poor communication with healthcare team, lack of family support, lack of knowledge, misconceptions, and limited health literacy. The results of the present study provided modifiable and nonmodifiable factors affecting treatment adherence among patients with type 2 diabetes. Modifiable factors are essential by performing appropriate interventions with the target group and health professionals.

## 1. Introduction

According to the World Health Organization (WHO), approximately 422 million people worldwide suffer from diabetes, with the Eastern Mediterranean countries having the highest prevalence (43 million) [1]. The International Diabetes Federation predicts that the prevalence of type 2 diabetes in Iran will increase from 9.6% in 2019 to 10.6% in 2030 and 11.1% in 2045 [2]. Treatment adherence refers to prevention of complications and successful control of diabetes, which are possible only with the active participation of patients in their treatment plans as recommended by their healthcare providers [3]. The definition of adherence in chronic conditions, as given by the WHO, is behaviors such as taking medications, following a diet, living a healthy lifestyle that correspond with recommendations of healthcare providers [4]. Treatment adherence has become a major challenge for medical professionals and social scientists. Efforts by physicians and healthcare professionals to ensure that patients adhere to medication regimens have frequently proven ineffective [5]. While some programs are aimed at increasing treatment adherence have been successful, it appears that approximately half of them have failed [6]. Patients' nonadherence to medication and diet regimens has significant economic and therapeutic consequences. For example, patients who do not adhere to medication are at risk for complications that compromise their health and severely affect their overall quality of life [7]. Treatment nonadherence has a negative influence on patients and results in the disease progressing and becoming more chronic throughout the world, so chronically ill patients need more treatment adherence [8].

Nonadherence to medication regimen is a complex behavioral process and is affected by several factors such as patients' characteristics, physician-patient interaction, and healthcare system [4]. The World Health Organization has divided the various factors affecting medication nonadherence into five categories: social and economic factors, healthcare team- and system-related factors, condition-related factors, therapy-related factors, and patient-related factors. Although some of these factors are unchangeable, patient-related factors can be changed by training and increasing their knowledge [9].

Health literacy is one of the variables affecting patients' knowledge of their disease [10]. Health literacy includes reading, listening, analytical, decision-making skills, and the ability to apply them to health situations [11]. The World Health Organization has identified health literacy as one of the biggest determinants of health. It has also recommended the countries around world to establish an association consisting of all affected people to monitor and coordinate strategic activities regarding the health promotion in different communities [12].

Many unpleasant health-related consequences occur because of poor health literacy. Limited health literacy has been associated with poor health outcomes, detrimental health behaviors, lower patient satisfaction, and in some cases, higher mortality [13]. Individuals with limited health literacy have trouble understanding the oral and written information provided by physicians, nurses, and insurers, as well as medication instructions, so they cannot achieve the health services they need in healthcare systems. These individuals have little knowledge about healthcare conditions and use less preventive services [14]. Studies show that health literacy is one of the factors influencing treatment adherence among patients with diabetes [15–17]. Limited health literacy is very common among patients with diabetes, which is associated with poor awareness and less understanding of diabetes as well as outcomes such as retinopathy and poor blood sugar control [18]. A practical intervention can improve knowledge, skills, attitudes, values, and behaviors. Education, by increasing knowledge and information, can create the necessary skills to achieve goals [19].

Many studies have examined the barriers to treatment adherence from different dimensions and with different inclusion criteria, so summarizing the findings of these studies can provide appropriate and correct strategies to increase treatment adherence among patients with type 2 diabetes. Therefore, the present study is aimed at reviewing the factors affecting the poor treatment adherence among patients with type 2 diabetes and limited health literacy in 2021. In addition, interventions can be designed, implemented, and evaluated to take an effective step in improving the health and quality of life of these patients.

## 2. Materials and Methods

This scoping review includes five stages including designing a research question, searching and extracting related studies, selecting related studies tabulating, and summarizing information and data and reporting results [20].

After the research question was designed (what factors affect the poor treatment adherence among patients with type 2 diabetes and limited health literacy?), a search strategy was developed, inclusion criteria were determined for selected studies, data extraction forms were prepared, and the data analysis program was identified.

### 2.1. Information Resources and Searches

The researcher searched Iranian (MagIran, SID, IranDoc, and IranMedex) and foreign databases (Embase, Science Direct, PubMed, Google Scholar, Scopus, and Web of Science) from 25 October 2021 to 14 November 2021. The search keywords were “Type 2 diabetes”, “barriers”, “treatment”, “medication”, “adherence”, “non-adherence”, “limited adherence”, and “limited health literacy”. Persian language databases were also searched. The definition of HL used in this review was developed by Kooshyar et al. [21]. This definition includes concepts such as numeracy, health education, health promotion, patient understanding, and comprehension. Moreover, the definition of treatment adherence developed by Mehrtak et.al was used in this review [22]. This definition includes concepts such as medication, diet, and lifestyle adjustments in accordance with the recommendations accepted by health care personnel.

In this study, the publication bias, source, and geography were investigated. In this study, these biases have not been done.

### 2.2. Inclusion and Exclusion Criteria

All qualitative, cross-sectional, descriptive-analytical, systematic, trial, and review articles addressed at least one of the factors influencing low treatment adherence from the perspective of patients, their families, or healthcare providers; the full texts of articles related to limited health literacy, which were published in valid journals from January 2010 to November 2021, were among inclusion criteria. Articles that did not specifically address the factors affecting treatment adherence among patients with diabetes and studies conducted before January 2010 were excluded.

### 2.3. Selection of Related Studies

Totally, 146 articles were obtained using the above keywords. Endnote was used to organize the studies. Duplicate studies were excluded. Then, after the titles and abstracts were reviewed, noneligible studies were eliminated. Therefore, the full texts of 63 studies were studied. At this stage, 45 articles were deleted, and finally, 18 articles published from 2013 to 2021 were selected and reviewed.

Data were extracted using a standard form and included the following categories: study IDs (study author and year of publication), location, study population, study type, sample size, main aims, materials and methods, theoretical approaches, results, and conclusions.

## 3. Results

Eighteen studies involving 3925 patients with T2DM from eight countries were included.

Measures of treatment adherence in these studies included Tiv et al. Medication Adherence questionnaire, Morisky Medication Adherence Scale (MMAS), the researcher-conducted dietary adherence questionnaire, the Brief Adherence Rating Scale (BARS), Adherence to Refills and Medicines Scale for Diabetes (ARMS-D), and Morisky Medication-Taking Adherence Scale (MMAS-4). HL measurement tools included Short-Test of Functional Health Literacy in Adults (S-TOFHLA), Test of Functional Health Literacy in Adults (TOFHLA), Literacy Assessment for Diabetes (LDA), the Diabetic Numeracy Test (DNT), Health Literacy Scale (HLS-EU-Q47), Newest Vital Sign (NVS), and the Brief Health Literacy Screen (BHLS).

According to the results of the study, four main areas of economic problems, poor communication of healthcare team, socioindividual factors, and limited health literacy were mentioned as barriers to treatment adherence (Table 1).

1. Economic problems

Studies have shown that financial problems are among the barriers to adherence to treatment. Adherence to a proper diet, physical activity, medication, blood sugar measurement, blood glucose meter (glucometer), and proper foot care is costly; and financial problems lead to the waiver of some self-care behaviors. Medical costs are a deterrent to continuing treatment in patients with diabetes, especially for patients with low economic status and patients without health insurance coverage [4, 23, 24].

(2) Poor communication of healthcare team

Poor communication of healthcare team was another factor in the treatment nonadherence among patients with diabetes. The results of the present study showed that the physician did not pay attention to the patient's words and did not behave well with patients. As patients cannot communicate continuously and usefully with their doctors, they stop continuing to see a doctor or reduce the number of their visits. Patients need counselling and interaction with the healthcare team, the necessary recommendations for disease management and motivation to adhere to the treatment [3]. Sometimes, doctors' improper behavior and attitude make patients unmotivated, so they stop their treatment. Therefore, the practice of the healthcare team is one of the most important factors in adhering to treatment among patients with diabetes. According to these results, training of communication techniques, especially effective listening skills, will play an effective role in this regard [25].

(3) Socioindividual factors

According to the findings of studies, some individual and social factors can play a role in adherence to treatment. These factors include patients' negative perceptions about the disease; worries about the side effects of drugs; lack of support from family, peers, and society; cultural and religious beliefs; inability to take drugs on a regular basis; forgetting doses; and experiencing drug side effects and disease [23, 24, 26]. Age and level of education were also mentioned as factors affecting treatment adherence [4].

(4) Limited health literacy

According to studies, although adherence to treatment is the most appropriate way to control type 2 diabetes, limited health literacy is an important barrier to patients' adherence to treatment instructions. Studies have shown that the level of health literacy in patients with type 2 diabetes is mostly low, and it is necessary to increase the level of health literacy in these patients by using strategies such as simplifying information or using simple and understandable training [21, 22].

Table 2 shows the full information of the articles and their results.

**Figure 1:**
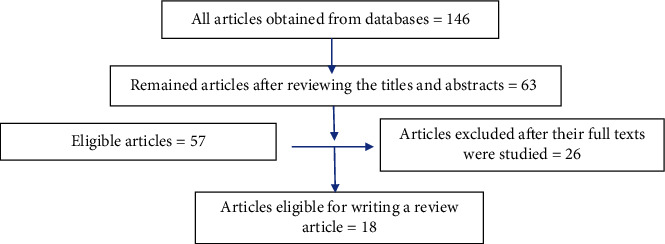
Flowchart of the selected articles.

The study flowchart is shown in Figure 1.

## 4. Discussion

The articles presented in this scoping review show modifiable and nonmodifiable factors affecting poor treatment adherence among patients with diabetes. Disbelief in the physicians' and healthcare providers' prescriptions; misconceptions about medications and diets; lack of support of family, peers, and community; complex treatment regimens; poor healthcare system; poor information and knowledge, concern, fear, discomfort, fatigue, and burnout; prioritization of other family members; poor working conditions; the healthcare team's poor practice, low self-efficacy, depression, multiple drug use, and low health literacy are among the modifiable factors affecting poor treatment adherence among patients with type 2 diabetes. According to the results of studies, the patient's dissatisfaction with working condition as well as reaction, behavior, and support of others and family prevents them from the treatment adherence [23, 34]. In addition, not following the diet due to poor working conditions, not having enough time to eat at workplace, having difficulty in treatment compliance due to the type of job, and forgetting to take medication due to high workload and stress are among the factors that prevent treatment adherence among patients [38]. Various studies considered the importance of others' support from such patients. Family members play an important role in patients' treatment adherence without whom adherence to the treatment regimen would be difficult and sometimes impossible [39]. Additionally, fear of judgment, compassionate behavior of others, and blaming the patient for the disease are factors that lead to poor treatment adherence among patients [38].

According to these results, the health care team's poor practice and patients' inability to communicate with health care providers are other barriers to adherence to treatment. Health care providers are unable to facilitate treatment adherence among patients with diabetes. They focus more on patients' clinical care and treatment, less on education and counselling, and do not have enough time to listen to patients' problems and train patients and their families, while patients demand a coordinated care or healthcare interactions, which are accompanied by discussion and understanding. Clinical decisions should be participatory, taking into account the individual preferences of patients and providing alternative treatment options to them. Other studies mentioned lack of training and counselling as one of the major barriers to diabetes management [3, 23, 27, 32]. Healthcare providers should simplify the treatment regimen (for example, reducing the number of times receiving medications on a day), negotiate with the patient about the priorities of the treatment regimen, remind care and appointments with follow-up programs, organize a care plan, design realistic goals in order to increase patients' cooperation and reduce the experience of failure, and encourage and reward the patient and family members to follow the treatment regimen or design appropriate rules in medical centres. In addition, patients should become familiar with diabetes associations or charities for their training [3].

The studies mentioned limited health literacy as an important barrier to treatment adherence. Health literacy is not simply the ability to read, rather it requires a complex group of reading, listening, analytical, and decision-making skills and the ability to apply these skills to health situations [40]. Health literacy helps a patient read, understand, recall, and follow health instructions [24]. High level of health literacy delays the disease complications, which is why health literacy can change behavior and lifestyle and promote health [41]. Ahmad et al. showed that for every one percent decrease in knowledge about the disease, treatment nonadherence had a 3.6-point increase [42]. Although treatment adherence is the most appropriate way to control type 2 diabetes, inadequate health literacy is an important barrier to patients' adherence to treatment [21, 26, 29, 33]. As a result, inadequate health literacy among these patients is a warning signal to health officials and policy makers and health care providers. Therefore, health promotion programs should pay more attention to health literacy. To increase health literacy, in addition to using simple and understandable educational materials, health education professionals' help is an effective measure to improve health literacy among patients with type 2 diabetes [22].

Some factors affecting treatment adherence among patients with diabetes can be modified by training and empowering patients and their families while some others by training healthcare providers, so the impact of these factors should be considered when designing educational interventions.

Nonmodifiable factors include economic problems, lack of insulin, age, education, cultural and religious beliefs, and the experience of some drug side effects. According to the results of the articles, financial problems have a major contribution to low treatment adherence among patients with diabetes. Following a proper diet and exercise, preparing medication, measuring blood sugar, preparing glucometer, and providing proper foot care are costly, so financial problems lead to the prevention of some self-care behaviors [24]. Healthcare costs are a deterrent to treatment adherence among patients with diabetes, especially patients with low socioeconomic status and patients without health insurance coverage [30]. According to the New Mexico Diabetes Prevention Program, low annual income and lack of health insurance were the main barriers to treatment adherence among patients with diabetes because 64% of the patients without insurance coverage and 6% of the patients with health insurance did not follow up their treatment status. Nam et al. reported that some patients with diabetes split their pills at each meal to reduce their treatment costs [43]. However, introducing and referring patients to charities and NGOs [3] can solve some of their financial problems, but this issue needs more detailed and comprehensive investigation. Lack of insulin is another factor that affects treatment adherence. Habte et al. found that patients unable to prepare their own insulin medication had difficulty adhering to their treatment [28]. Some studies mentioned age and education as factors influencing treatment nonadherence. However, Nelson et al. demonstrated that younger age was an obstacle to treatment adherence [26], while Gholamaliei et al. considered older age as an obstacle to treatment adherence [4]. However, further studies are required to achieve an accurate and logical result.

Cultural and religious beliefs are other factors that may prevent people from adhering to treatment. Mukona et al. demonstrated that sometimes families obliged patients to consult with traditional and religious therapists. Some patients reported seeking help from religious healers because they believed their illness was linked to demons and the curse of generations. On the other hand, consultation with traditional and religious therapists was in some cases cheaper or free, which was attractive to families, especially when the patient had no income. Some participants used herbs to control blood sugar because insulin was expensive and difficult to store. Researchers have found that there may be a correlation between financial problems and cultural and religious beliefs [23]. It should be noted that some cultural and religious beliefs rooted in superstitions and misconceptions may be eliminated by awareness or education, but some other cultural and religious beliefs are too deep to remove. However, there is a need for the relevant authorities to try to change people's misconceptions.

Studies have reported the experience of drug side effects as a barrier to treatment [24]. The side effects of diabetes medications, especially insulin, are unavoidable and may occur in patients who inject insulin [44]. This issue becomes more important when individuals do not adhere to their medication regimen well and changes their doses.

The results of this study can be used to design comprehensive programs to empower patients with type 2 diabetes in treatment adherence by introducing different factors of barriers to treatment adherence among patients with type 2 diabetes.

One of the limitations of this study is that although eligible articles were identified and reviewed, some unpublished studies might have been missed. In addition, as the present study is a scoping review, there was no limitation for selecting articles; therefore, the quality of the articles did not assess. This may affect the study results.

According to the results of this study, it is suggested that educational and counselling services be provided to increase awareness, promote health literacy of patients with diabetes, and motivate and encourage them to follow treatment regimens in order to increase their health and quality of life. Policymakers and planners are also recommended to improve the physical, mental, and social health of patients with type 2 diabetes and their families by designing and implementing educational interventions. Further studies are suggested to examine the effectiveness of educational interventions in promoting treatment adherence and limited health literacy among patients with type 2 diabetes.

## 5. Conclusions

The results of the present study provided modifiable and nonmodifiable factors affecting treatment adherence among patients with type 2 diabetes. As most of these factors can be modified, it is possible to take an important step towards empowering patients by implementing targeted educational interventions. Since some of these factors refer to the role of patients' families and health care teams, in addition to empowering and training patients, their families and health care providers also need to be trained to implement treatment adherence among patients with type 2 diabetes.

## Figures and Tables

**Table 1:** The main results of this study.

Barriers to treatment adherence among patients with type 2 diabetes
Economic problems
Poor communication of healthcare team
Socioindividual factors
Limited health literacy

**Table 2:** Studies on factors affecting poor treatment adherence among patients with type 2 diabetes with limited health literacy.

Author	Country and language	Study type	Main aim	Sample size	Method	Result
Rezaei et al. (2019) [27]	Iran (English)	Qualitative	Determining barriers to treatment adherence among patients with type 2 diabetes	*N* = 12	This study was conducted using content analysis method. Semistructured interviews were used to collect data.	Barriers to treatment adherence included four main categories: disbelief in descriptive/prescriptive medical knowledge, personal illness experiences, daily life challenges, and interactive/economic challenges.
Habte et al. (2017) [28]	Ethiopia	Qualitative	Determining barriers and facilitators of adherence to diabetes medications in patients with diabetes	*N* = 39	This study was conducted purposefully. Open coding was used to analyze the data and to identify key topics.	In this study, patients' negative perceptions of the disease, concerns about the side effects of medications, lack of insulin, and the practice of healthcare providers were considered as barriers to treatment adherence.
Hussain et al. (2020) [29]	Pakistan (English)	Cross-sectional	Studying the effect of health literacy on treatment adherence among older women with type 2 diabetes	*N* = 524	This study was conducted using convenience sampling. All participants in the study completed test of health literacy, diabetic numeracy test, and medication adherence rating scale.	According to the study, people with good health literacy were more likely to remember to take their medications than those who had poor health literacy.
Mukona et al. (2017) [23]	Zimbabwe, South Africa (English)	Qualitative	Determining adherence barriers and possible solutions for nonadherence to diabetes treatment among women with gestational diabetes: patients' perspectives	*N* = 28	This study was conducted on pregnant women aged 18-49 years. Each participant completed a semistructured questionnaire. Data were analyzed thematically and manually.	Barriers mentioned in this study included poor socioeconomic status; lack of family, peer, and community support; pregnancy effects; complex treatment regimen; pathophysiology of diabetes; cultural and religious beliefs; and a poor health care system.
Alwazae et al. (2019) [30]	Saudi Arabia (English)	Cross-sectional	Barriers to treatment adherence to diabetic retinopathy in Saudi adults	*N* = 404	A five-part questionnaire was used to collect data: demographic data, diabetes index, assessment of knowledge about DR, attitudes towards DRS, and DRS barriers. Data were analyzed using SPSS 23.	The results showed that poor knowledge and financial problems were obstacles to treatment adherence.
Abdullah et al. (2020) [31]	Malaysia (English)	Cross-sectional	Prevalence of limited health literacy and its related factors among patients with type 2 diabetes	—	This cross-sectional study was conducted from January to March 2018. Health literacy level was measured using the HLS-EU-Q47.	According to the results of this study, the prevalence of limited health literacy is high among patients with type 2 diabetes in Perak, Malaysia.
Huang and Shiyanbola (2021) [32]	The US (English)	Cross-sectional	Evaluation of barriers and facilitators of treatment adherence among patients with type 2 diabetes and different levels of health literacy	*N* = 228	In this mix methods study, 205 participants completed the survey questionnaire and 23 participated in the semistructured interviews.	A sense of over-control by taking diabetes medications, inability to take medications regularly, distrust in providers, concerns about drug safety, and ambiguity about the role of medications were some of the barriers to treatment adherence.
Fan et al. (2016) [33]	The US (English)	Cross-sectional	Studying the relationship between health literacy and unintentional and intentional nonadherence to treatment among patients with type 2 diabetes	*N* = 208	Information was obtained from a written questionnaire and the patient's medical record. Bivariate and multivariate regressions were used to investigate the predictors of medication nonadherence.	The results of this study showed that limited health literacy was significantly associated with increased unintentional nonadherence to treatment, but was not associated with intentional nonadherence.
Nelson et al. (2018) [26]	The US (English)	Randomized controlled trial	Evaluation of barriers to adherence to diabetes treatment using information-motivation-behavioral skill model (IMB)	*N* = 237	The checklist identifying barriers to adherence and HbA1c was completed for them. The most common adherence barriers were identified and the relationship between patient characteristics and barriers reported on each of the IMB constructs were examined.	Forgetting doses, lower age, and poor health literacy were among the most reported barriers.
Mostafavi et al. (2021) [34]	Iran (English)	Qualitative	Determining psychosocial barriers to treatment adherence among patients with type 2 diabetes	*N* = 23	This qualitative study was conducted in Isfahan. Participants were interviewed face to face from November 2017 to June 2018. Data were analyzed using MAXQDA-10 and content analysis.	Data analysis revealed six categories of psychosocial barriers affecting treatment adherence: (1) fear, concern, and discomfort; (2) fatigue and burnout; (3) prioritization of children's issues; (4) poor financial support; (5) communication challenges; and (6) improper work conditions.
Mousavizadeh et al. (2016) [3]	Iran (Persian)	Qualitative	Determining barriers to treatment adherence among patients with diabetes	*N* = 15	This study conducted semistructured in-depth interviews. Data were collected from December 2015 to July 2016 and analyzed by contemporary content analysis.	The results showed that the three main themes of poor practice of the healthcare team, social limitations, and personal helplessness were identified as barriers to treatment adherence.
Gholamaliei et al. (2016) [4]	Iran (Persian)	Cross-sectional	Evaluation of medication adherence and its related factors among patients with type 2 diabetes	*N* = 300	The research instruments included a researcher-made questionnaire related to the factors affecting medication adherence and a questionnaire measuring the degree of medication adherence. SPSS19 was used for data analysis.	The results of the present study showed that age, level of education, healthcare cost, healthcare team and health system costs, factors related to disease treatment and status, beliefs related to the disease, self-efficacy, and concerns about medication taking were factors affecting treatment adherence.
Kooshyar et al. (2014) [21]	Iran (Persian)	Descriptive-analytical	Investigating the relationship between health literacy, adherence to treatment regimen, and quality of life related to health among older adults with diabetes	*N* = 300	Cluster sampling was used. Data were collected using the brief-TOFHL, health-related quality of life, MMAS, a researcher-made tool for diet, and exercise compliance, as well as HbA1C and BMI measurements.	According to the results, inadequate health literacy has a direct effect on adherence to the treatment regimen in older adults.
Dehvan et al. (2018) [24]	Iran (Persian)	Integrative review	Determination of inhibitors and facilitators of adherence to treatment regimens among patients with type 2 diabetes	—	In this review study, all full-text Persian and English articles (from 2000 onwards) on adherence to treatment regimens among patients with type 2 diabetes were reviewed, and finally, 53 articles were included in the study.	Depression, financial problems, drug side effects and illness, memory problems, simultaneous use of several drugs, and the complexity of the treatment regimen were the most important barriers to adherence to the treatment regimen in this study.
Hashemi & Bouya et al. (2018) [35]	Iran (Persian)	Review	Adherence to treatment among patients with diabetes: an important but neglected issue	—	—	The most important barriers to treatment adherence are poor practice of the healthcare team, social limitations, and personal helplessness.
Mehrtak et al. (2017) [22]	Iran (Persian)	Cross-sectional	Evaluation of the relationship between health literacy, adherence to medication, nutrition, and exercise among patients with type 2 diabetes	*N* = 241	This study was conducted by random sampling. Data were collected by TOFHLA, MMAS-8, and adherence to diet and exercise questionnaire.	According to the results of this study, the level of health literacy affects the proper adherence to medication, nutrition, and exercise among patients with type 2 diabetes.
Bauer et al. (2013) [36]	California (English)	Cross-sectional	Evaluation of health literacy and adherence to antidepressants among adults with diabetes	*N* = 1366	Adults with type 2 diabetes who completed a survey in 2006 participated in the study. The accredited self-report scale measured health literacy.	According to the results, patients with limited health literacy had poor adherence compared to patients with good health literacy.
Brundisini et al. (2015) [37]	Canada (English)	Qualitative meta-synthesis	Examining the different views of patients with type 2 diabetes and providers about nonadherence to the medications	*N* = 86	This study reviewed published articles between 2002 and 2013. Eighty-six studies were eligible for coding and thematic analysis	The results of this study identified 7 categories of barriers: (1) emotional experiences as positive and negative stimuli for adherence; (2) intentional noncompliance; (3) patient-provider relationship; (4) information and knowledge; (5) prescription of medicine; (6) social and cultural beliefs; and (7) financial problems.
